# *APOE* ε4, Alzheimer’s disease neuropathology and sleep disturbance, in individuals with and without dementia

**DOI:** 10.1186/s13195-022-00992-y

**Published:** 2022-03-30

**Authors:** Jonathan Blackman, Seth Love, Lindsey Sinclair, Richard Cain, Elizabeth Coulthard

**Affiliations:** 1 North Bristol NHS Trust, Bristol, UK; 2 Learning and Research, University of Bristol, Southmead, Bristol, BS10 5NB UK

**Keywords:** Alzheimer’s, Dementia, Sleep, APOE-E4, Apolipoprotein, Cognitive impairment

## Abstract

**Background:**

Apolipoprotein E epsilon 4 (*APOE*-ε4) carrier status is an established risk factor for Alzheimer’s disease (AD) dementia. It has also been linked with sleep disturbance in healthy older adults and increased insomnia risk. This association may be driven by the effect of *APOE*-ε4 on AD pathological change, itself associated with sleep abnormalities. To assess this relationship, we have evaluated post-mortem neuropathological findings in patients with and without cognitive impairment and AD pathology, who had extensive clinical assessment within 12 months of death.

**Methods:**

This retrospective cohort study used UK Brain Banks Network data. Eligible subjects were aged over 50, with pre-mortem neuropsychiatry inventory scores of sleep disturbance (NPI-K), neurocognitive testing and functional cognitive status assessment (Clinical Dementia Rating scale). Neuropathological data included Thal phase, Braak stage and CERAD scores (measures of Aβ plaque distribution, tangle distribution and neuritic plaque density, respectively) combined to form the National Institute on Aging Alzheimer’s Association (NIA-AA) ABC score reflecting AD neuropathology. Participants with other significant intracerebral pathology or pathological features of non-AD dementia were excluded.

Multivariate linear regression was performed with NPIK Global Score (NPIK frequency score multiplied by severity score) as the dependent variable and *APOE*-ε4 heterozygosity or homozygosity as independent variables. Covariates included age, gender, *APOE*-ε2 status and ABC NPI measures reflecting depression and anxiety. Further models stratified by ABC score and functional cognitive status were also produced.

**Results:**

Seven hundred twenty-eight records were identified. Two hundred two participants were included in the final analysis: mean (SD) age 84.0 (9.2) and MMSE 14.0 (11.8). Mean sleep disturbance scores were highest in ε4 homozygosity (*n*=11), 4.55 (5.4); intermediate in ε4 heterozygosity (*n*=95), 2.03 (4.0); and lowest in non-ε4 carriers (*n*=96), 1.36 (3.3). Within the full sample, controlling for pathological status, age, gender, depression, anxiety and CDR-SOB status, *APOE*-ε4 homozygosity was associated with sleep disturbance (*β* 2.53, *p*=0.034). *APOE-*ε4 heterozygosity was similarly associated in individuals without dementia (*β* 1.21, *p*=0.048).

**Conclusion:**

These findings lend weight to the hypothesis that *APOE*-ε4 affects sleep by mechanisms independent of AD pathological change. Evaluation of those mechanisms would enhance understanding of sleep disturbance pathways and potentially provide treatment targets.

**Supplementary Information:**

The online version contains supplementary material available at 10.1186/s13195-022-00992-y.

## Background

Relative to the common ε3 allele of apolipoprotein E (*APOE* ε3), the ε4 allele is an established risk factor for the development of sporadic and late-onset familial Alzheimer’s disease (AD) [1–3]. Within predominantly Caucasian populations, increasing allele dose is positively associated with AD risk, with ε4 heterozygosity conferring an odds ratio (OR) of approximately 3 and ε4 homozygosity an OR of approximately 14 [4]. *APOE* ε4 also lowers the age of onset in a similarly allele-number-dependent manner, with one allele advancing onset by 2–5 years and two by 5–10 years [5] although such relationships appear weaker in African-American populations [6–8].

Multiple mechanisms have been proposed through which *APOE* ε4 may exert these effects [9]. Apolipoprotein E4 (ApoE4) affects amyloid beta (Aβ) metabolism, predisposing to its extracellular deposition as amyloid plaques [10–12] and to more severe cerebral amyloid angiopathy [13–16]. Whilst effects on Aβ are hypothesised to represent the dominant pathway, proteolytic ApoE4 cleavage resulting from stress or injury also predisposes to tau hyperphosphorylation and neurofibrillary tangle (NFT) formation [17]. ApoE4 has further been associated with disruption to glucose metabolism [18–20], blood-brain barrier integrity [21], cerebrovascular function [22], lipid transport [23], synaptic function [24] and inflammatory responses [25] as well as neuronal toxicity and α-synuclein/TDP-43 pathologies [26, 27].

Multiple studies have also linked *APOE* ε4 to sleep disturbance: specifically, objective sleep disturbance in healthy older adults [28], an increased risk of insomnia [29] and obstructive sleep apnoea/sleep-disordered breathing in both adults [30, 31] and children [32]. Improved sleep was reported to attenuate the negative effect of ε4 on incident AD [33]. Additionally, ε4 has been proposed as a mediator of the relationship between sleep and cognitive decline, both obstructive sleep apnoea (OSA) and *APOE* ε4 impairing cognitive performance [34–36]. However, whilst possession of ε2 reduces the odds of developing AD [37, 38], this allele has been linked with increased likelihood of OSA [39].

Sleep disturbance, whilst traditionally associated with established AD disease [40–42], is detectable prior to the emergence of symptoms [43–45] and plays a potentially causative role in AD pathogenesis [46–48]. Therefore, *APOE* ε4 could influence AD incidence and progression through the effects of this allele on sleep. Establishing this categorically is complicated by the influence of ApoE4 on the pathological hallmarks of AD themselves detectable decades prior to symptomatic presentation with cognitive impairment [49] and also associated with sleep disturbance. Hippocampal and Entorhinal Cortex deposition of NFTs found in early Braak stages [50] have been associated with an increased likelihood of sleep disturbance [51]. AD pathology within the suprachiasmatic nuclei and the ventrolateral preoptic area has also been implicated in sleep disturbance [52, 53] with hippocampal Aβ burden in otherwise healthy adults correlating significantly with impairments in non-random eye movement (NREM) slow wave activity generation and showed a trend towards deterioration in macro-architectural sleep parameters [54].

Here, we have tested the hypothesis that *APOE* ε4 allele count increases sleep disturbance in people with and without cognitive impairment, independently of its influence on the two major hallmark AD pathologies (Aβ plaques and tau neurofibrillary tangles). We have controlled for the extent of AD pathological change by the gold standard of post-mortem neuropathological assessment [55] according the 2012 National Institute on Aging-Alzheimer’s Association Guidelines [56] and have excluded individuals with other significant intracerebral pathology.

## Methods

### Participants

This retrospective cohort study used data obtained for participants in the Brains for Dementia Research (BDR) Programme (https://www.brainsfordementiaresearch.org.uk/) and held on the UK Brain Banks Network (UKBBN) database. The database holds demographic and neuropathological details of donated brains, processed and assessed according to detailed and comprehensive post-mortem protocols, as well as clinical assessments undertaken prior to post-mortem as part of the BDR project established in 2007. This links 5 brain banks across the UK (London, Oxford, Newcastle, Bristol and Manchester) with common protocols for consent, tissue handling and quality indicators. Volunteers were recruited via posters, radio adverts, presentations to groups/clubs and signposted via Alzheimer’s Research UK and Alzheimer’s Society charities. The population comprises many healthy participants with a positive family history of dementia and also participants with a diagnosis of dementia [57]. See Table 1 for inclusion and exclusion criteria.

**Table 1:** Inclusion and exclusion criteria

Inclusion criteria	Exclusion criteria
UK Brain Bank Network Participant Recorded Braak, Thal and CERAD pathological stage Each of the following within 12 months pre-mortem • *Neuropsychiatry Inventory K measure of sleep disturbance* • *Clinical Dementia Rating (CDR) global score* Age > 50	Significant intracerebral pathology other than Alzheimer’s disease including the following: • *Severe cerebrovascular disease (ischaemic and haemorrhagic)* • *Lewy body disease as defined by Braak LB stage > 0* • *Neoplasia, epilepsy, CNS inflammation, trauma, vascular malformation, prion disease* Pathological findings in keeping with dementia other than Alzheimer’s dementia including Frontotemporal dementia, vascular dementia and Lewy body dementias

A spectrum of histopathological findings is represented, ranging from healthy tissue to marked AD neuropathological changes, in individuals both with and without clinical AD dementia. Included participants had full *APOE* genotyping.

### Outcome measure

Sleep disturbance was measured by component K of the neuropsychiatric inventory (NPI-K) [58]. These score responses provided by an informant, caregiver or study partner including increased latency, increased wake time after sleep onset, wandering, early morning wakening, excessive daytime sleep and sleep-wake cycle disturbance. A global score was obtained by multiplying frequency and severity domains (see Table 2).

**Table 2:** Neuropsychiatric inventory measure of sleep disturbance (NPI)

Frequency score			Severity score		
1	Occasionally	Less than once per week	1	Mild	Night-time behaviours occur but they are not particularly disruptive
2	Often	About once per week	2	Moderate	Night-time behaviours occur and disturb the participant and the sleep of the caregiver, more than one type of night-time behaviour may be present
3	Frequently	Several times per week but less than every day	3	Marked	Night-time behaviours occur; several types of night-time behaviour may be present; the participant is very distressed during the night and the caregiver’s sleep is markedly disturbed
4	Very frequently	Once or more per day			

### Neuropathologic data

Each participant had undergone post-mortem analysis of CERAD neuritic plaque stage, Thal Aβ plaque stage and Braak NFT stage allowing for calculation of the National Institute on Aging-Alzheimer’s Association ABC Score [56]. The combination of A, B and C score determine the extent of AD neuropathological change, designated “Not”, “Low”, “Intermediate” and “High”.

### Statistical analysis

For data cleaning and analysis, we used R Studio v3.6.3 statistical software. Raw scores were used throughout. Demographic and clinical variables were tested across groups for normality and compared made using Kruskal-Wallis and Pearson chi-squared tests. All tests of significance were two-tailed with *α* = 0.05.

Descriptive statistic was produced with unadjusted means of sleep disturbance by *APOE* ε4 status. These were calculated for the whole population before being stratified by NIAA-AA ABC score and CDR status. A fourth unadjusted comparison stratified the whole population into four phenotypically separate groups categorised by low CDR (0/0.5) or high CDR (1/2/3) and low ABC score (none/low) or high ABC score (intermediate/high). These groups are termed ‘Healthy’ (low CDR and low ABC), ‘Other Cognitive Impairment’ (high CDR and low ABC), ‘Alzheimer’s Disease’ (low CDR and high ABC) and ‘Alzheimer’s dementia’ (high CDR and high ABC).

The primary outcome measure was determined by multivariate linear regression with NPI-K Sleep Disturbance Global Score as the dependent variable. Crude and adjusted analyses were performed including dummy variables reflecting *APOE* ε4 allele copy number, with 0 as reference. Covariates were introduced to an adjusted model to control for *APOE* ε2 allele number, age, gender, CDR sum of boxes (CDR-SOB) and neuropsychiatry inventory measures of depression and anxiety. Dummy variables were created to reflect NIAA-AA ABC neuropathological stages. All regression models were checked for multicollinearity with variance inflation factors < 10. The study is powered at 80% to detect an effect size of *f*^2^ = 0.088 (*n* = 202, *α* = 0.05).

As a post hoc sensitivity analysis, these regressions were repeated in groups stratified by NIAA-AA ABC score and CDR status.

## Results

### Participant selection

**Fig. 1:**
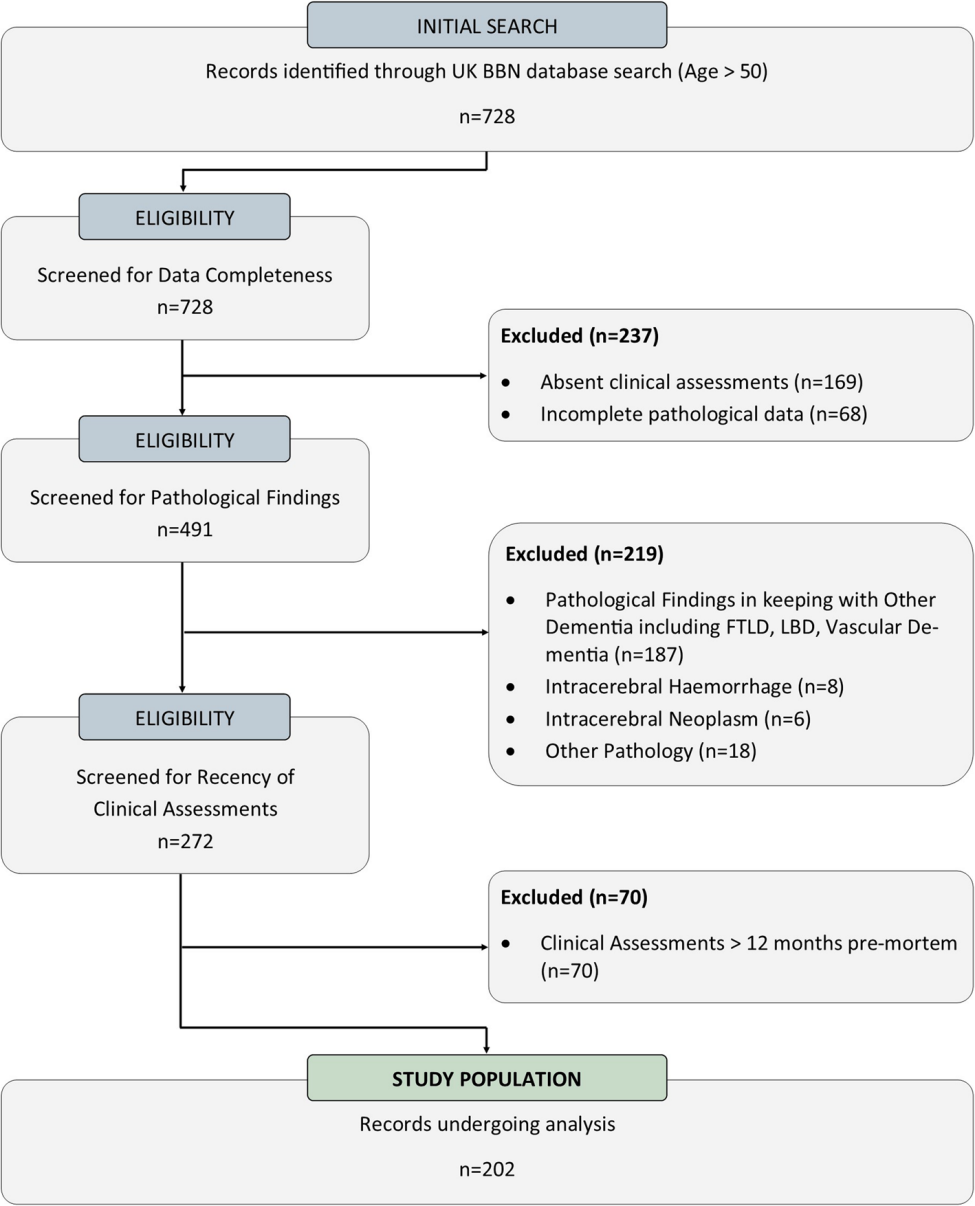
Participant flow diagram

Initial database search yielded *n* = 728 BDR cases, of which *n* = 202 fulfilled our criteria for analysis (See Fig. 1).

### Baseline demographics

Selected participants had a mean age of 84.0 years (SD = 9.2), 51.0% were male, mean CDR 1.8 (SD = 1.3) and mean Mini-Mental State Examination (MMSE) score 14.0 (SD = 11.8). Baseline demographics of the study population stratified by *APOE*-ε4 allele count (non-ε4 carriers *n* = 96, ε4 heterozygotes *n* = 95, ε4 homozygotes *n* = 11) are shown in Table 3. There were statistically significant differences in AD ABC stage, mean MMSE and mean CDR scores.

**Table 3:** Baseline demographics by APOE-ε4 status

	Non ε4 carrier *n*=96	ε4 heterozygosity *n*=95	ε4 homozygosity *n*=11	***p*** value
Mean age (SD)	85.2 (9.1)	83.0 (9.3)	81.5 (8.5)	0.162*
Gender male no. (%)	48 (50.0)	49 (51.6)	6 (54.5)	0.948†
ABC stage				
*None*	16	3	0	<0.001†
*Low*	41	18	0	
*Intermediate*	15	23	4	
*High*	24	51	7	
Mean MMSE (SD)	16.1 (12.5)	12.2 (11.0)	9.0 (9.8)	0.063*
Mean CDR Global Score (SD)	1.6 (1.3)	2.0 (1.2)	2.8 (0.8)	0.002*
Mean CDR SOB	8.8 (8.0)	12.2 (6.8)	16.2 (5.1)	<0.001*

*p values calculated by Kruskal-Wallis test

^†^Chi-squared Test

### Sleep disturbance by APOE ε4 status

Crude sleep disturbance scores in the cohort stratified by ε4 are shown in Table 4. There were statistically significant increases in all neuropsychiatry inventory measures of sleep disturbance between those with 2 vs 0 alleles. Severity, frequency and caregiver distress domains were also significantly higher in those with 2 vs 1 allele. There were increased caregiver distress scores only in those with 1 allele compared with 0.

**Table 4:** Neuropsychiatry inventory sleep disturbance scores by *APOE* ε4 status

	Non-ε4 carrier *n*=96	ε4 Heterozygosity *n*=95	ε4 Homozygosity *n*=11	***p*** value*		
				0 vs 1 ε4 Alleles	1 vs 2 ε4 Alleles	0 vs 2 ε4 Alleles
NPI K Global Score (SD)	1.36 (3.3)	2.03 (4.0)	4.55 (5.4)	0.236	0.107	0.024
NPI K Severity Score (SD)	0.42 (0.9)	0.57 (1.0)	1.27 (1.5)	0.135	0.034	0.013
NPI K Frequency Score (SD)	0.67 (1.4)	0.92 (1.6)	1.64 (1.9)	0.103	0.029	0.008
NPI K Caregiver Distress Score (SD)	0.39 (1.2)	0.53 (1.3)	1.00 (1.7)	0.003	0.007	<0.001

*p values calculated by Wilcoxon rank-sum test

**Fig. 2:**
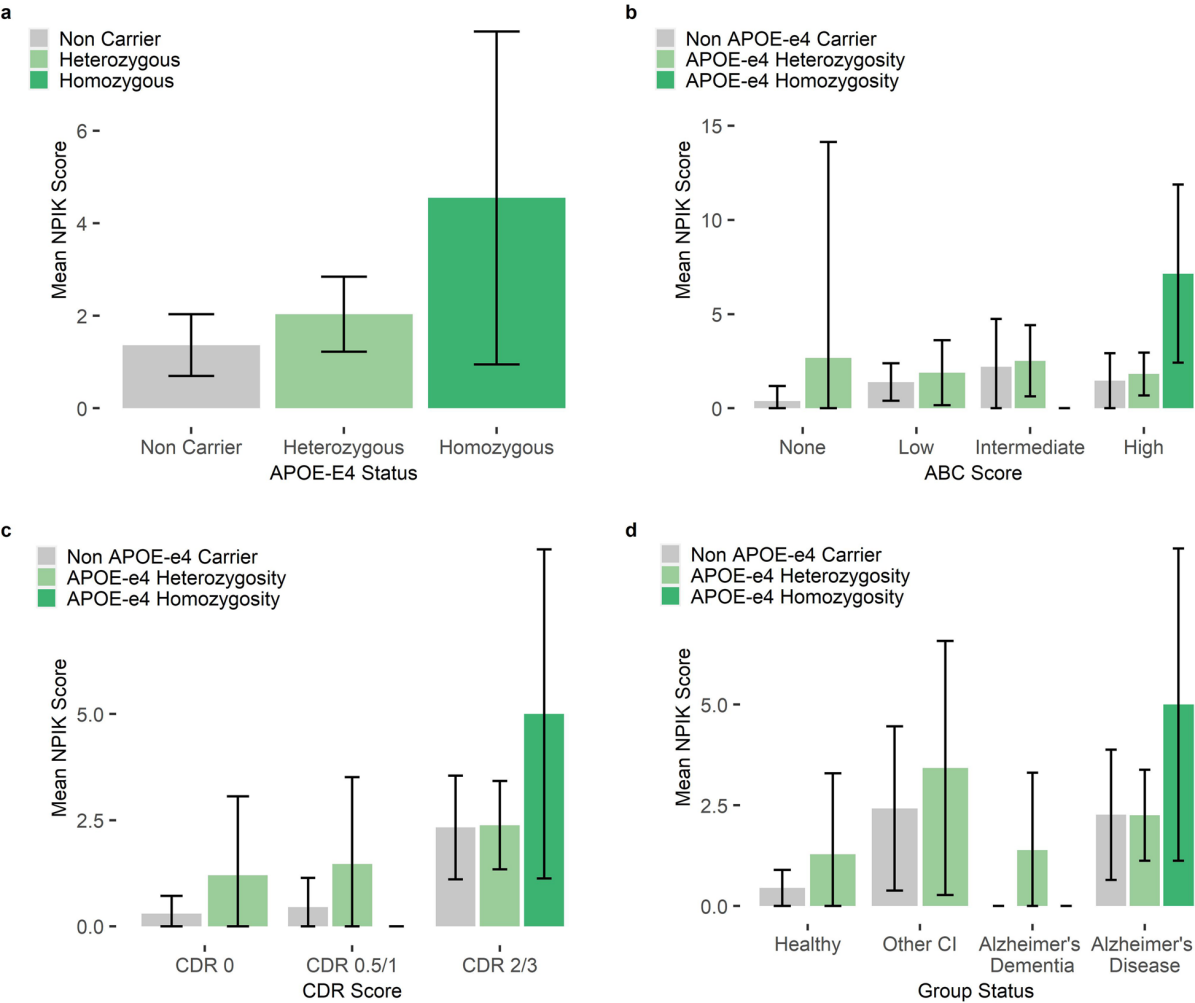
Unadjusted NPIK sleep disturbance scores by APOE-E4 status. **a** Shows unadjusted global sleep disturbance scores and 95% confidence intervals by APOE ε4 status across the full population. Unadjusted global sleep disturbance scores are presented for the population stratified by NIAA-AA ABC Score (**b**) and CDR score (**c**). For unadjusted sleep scores by group status (healthy, other cognitive impairment, Alzheimer’s disease and Alzheimer’s dementia), see (**d**)

Positive trends between ε4 allele number and increasing mean NPIK sleep disturbance score were across the full-cohort irrespective of stratification by CDR status, ABC score of neuropathological change and clinical classification (Fig. 2).

### Primary analysis

Full multivariate linear regression revealed a statistically significant effect of *APOE* ε4 homozygosity on global scores of sleep disturbance (*β* 2.53, *p*=0.034) controlling for AD pathological status, ε2 carrier status, age, gender, depression, anxiety and CDR-SOB status. A positive trend was found for ε4 heterozygosity in both crude (*β* 0.67, *p*=0.221) and adjusted (*β* 0.41, *p*=0.471) analyses, although neither reached statistical significance (see Table 5).

**Table 5:** NPIK Global Sleep Disturbance Score—full population linear regression

Full population *n*=202						
	Crude analysis			Adjusted analysis		
	*β*	SE	*p*	*β*	SE	*p*
*APOE* ε4 Status^a^						
*Heterozygous*	0.67	0.54	0.221	0.41	0.57	0.471
*Homozygous*	3.18	1.20	0.008	2.53	1.18	0.034
NIA-AA ABC Stage^b^						
*Low*				0.51	0.94	0.590
*Intermediate*				0.07	1.04	0.943
*High*				-0.73	1.05	0.493
*APOE* ε2 carrier status				1.43	1.24	0.252
Age				-0.08	0.03	0.004
Male gender				-0.53	0.50	0.293
NPI-D Global Score (depression)				0.01	0.14	0.928
NPI-E Global Score (anxiety)				0.19	0.13	0.145
CDR-SOB				0.13	0.04	0.003

^a^ Baseline Reference of APOE ε3/ε3, ε2/ε3, and ε2/ε2

^b^ Baseline reference of NIA-AA ABC Score ‘None’

Further, multivariate regression testing additional models were performed post hoc (Supplementary Material Table 1). Whilst the effect size estimates for *APOE* ε4 status within these models differed, overall trends and statistical significance remained unaltered. To further assess the independent effects of *APOE* ε4 status, further multivariate regressions were performed after stratification of the cohort by neuropathological change and CDR status (Tables 6 and 7). Positive trends between *APOE* ε4 status and sleep disturbance were seen in all stratified groups. Sleep disturbance was significantly associated with ε4 heterozygosity in the group without clinical dementia (CDR 0/0.5) (*β* 1.28, *p*=0.024) and with ε4 homozygosity in the relatively cognitively impaired group (CDR 1/2/3) (*β* 2.95, *p*=0.045).

**Table 6:** NPIK Sleep Disturbance Score—adjusted analysis stratified by neuropathological change^a^

	β	SE	*p*
ABC score 0/1 (“None”/“Low), *n*=80			
*APOE* ε4 status^b^			
*Heterozygosity*	0.97	0.75	0.200
*Homozygosity*	NA‡	NA‡	NA‡
ABC Score 2/3 (“Intermediate”/“High”), *n*=129			
*APOE* ε4 status^b^			
*Heterozygous*	0.42	0.83	0.609
*Homozygous*	2.85	1.40	0.045

^a^ Adjusted by age, gender, *APOE* ε2 status, NPI-D, NPI-E and CDR-SOB

^b^ Baseline reference *APOE* ε3/ε3, ε2/ε3 or ε2/ε2

^‡^No participants within the ABC 0/1 group with 2 *APOE* ε4 alleles

**Table 7:** NPIK Sleep Disturbance Score—adjusted analysis stratified by CDR status^a^

	*β*	SE	*p*
CDR score 0/0.5, *n*=67			
*APOE* ε4 status^b^			
*Heterozygous*	1.21	0.60	0.048
*Homozygous*	0.13	2.17	0.952
CDR score 1/2/3, *n*=142			
*APOE* ε4 status^b^			
*Heterozygous*	0.10	0.80	0.897
*Homozygous*	2.77	1.93	0.048

^a^Adjusted by age, gender, *APOE* ε2 status, NPI-D, NPI-E and NIA-AA ABC score

^b^Baseline reference *APOE* ε3/ε3, ε2/ε3 or ε2/ε2

## Discussion

In this large, community-based cohort, *APOE* ε4 homozygosity was independently associated with sleep disturbance after controlling for the extent of AD neuropathological change, age, gender and affective symptoms, in individuals both with and without dementia. Homozygosity conferred a 2.53 (±1.18) mean point increase in NPI-K global sleep disturbance score. A non-significant trend towards an increased score was also noted with *APOE* ε4 heterozygosity, which conferred a 0.41-mean point increase in sleep disturbance score. Within the group without dementia (CDR 0/0.5), ε4 heterozygosity conferred a statistically significant increase in sleep disturbance score of 1.21 points.

Understanding of the relationship between ApoE status and sleep disturbance continues to evolve. Shortening of rapid-eye-movement (REM) sleep in individuals with MCI was significantly more apparent in carriers than non-carriers of the ε4 allele [59]. However, such differences extend beyond populations affected by cognitive impairment. Objective sleep disturbance in healthy adults as measured by polysomnography and actigraphy was found to be independently associated with the presence of the ε4 allele [28]. Furthermore, in a study that controlled for demographic variables, the ε4 allele was associated with insomnia in those both with and without psychiatric disorders [29]. Improved sleep attenuated the increased risk of AD development conferred by possession of ε4, in particular modifying its effect on neurofibrillary tangle formation [33]. Conversely, ε4 carriers with dementia were found to have slower rates of sleep disturbance progression than non-carriers [60] albeit in a small cohort. Our findings support previous findings indicating that people with one or more *APOE* ε4 alleles are likely to have more impaired sleep but add to these findings by controlling for the severity of Alzheimer’s disease pathology as determined neuropathologically. There are a range of potential explanations for these findings:

### Apo E4-mediated AD pathological change

**Fig. 3:**
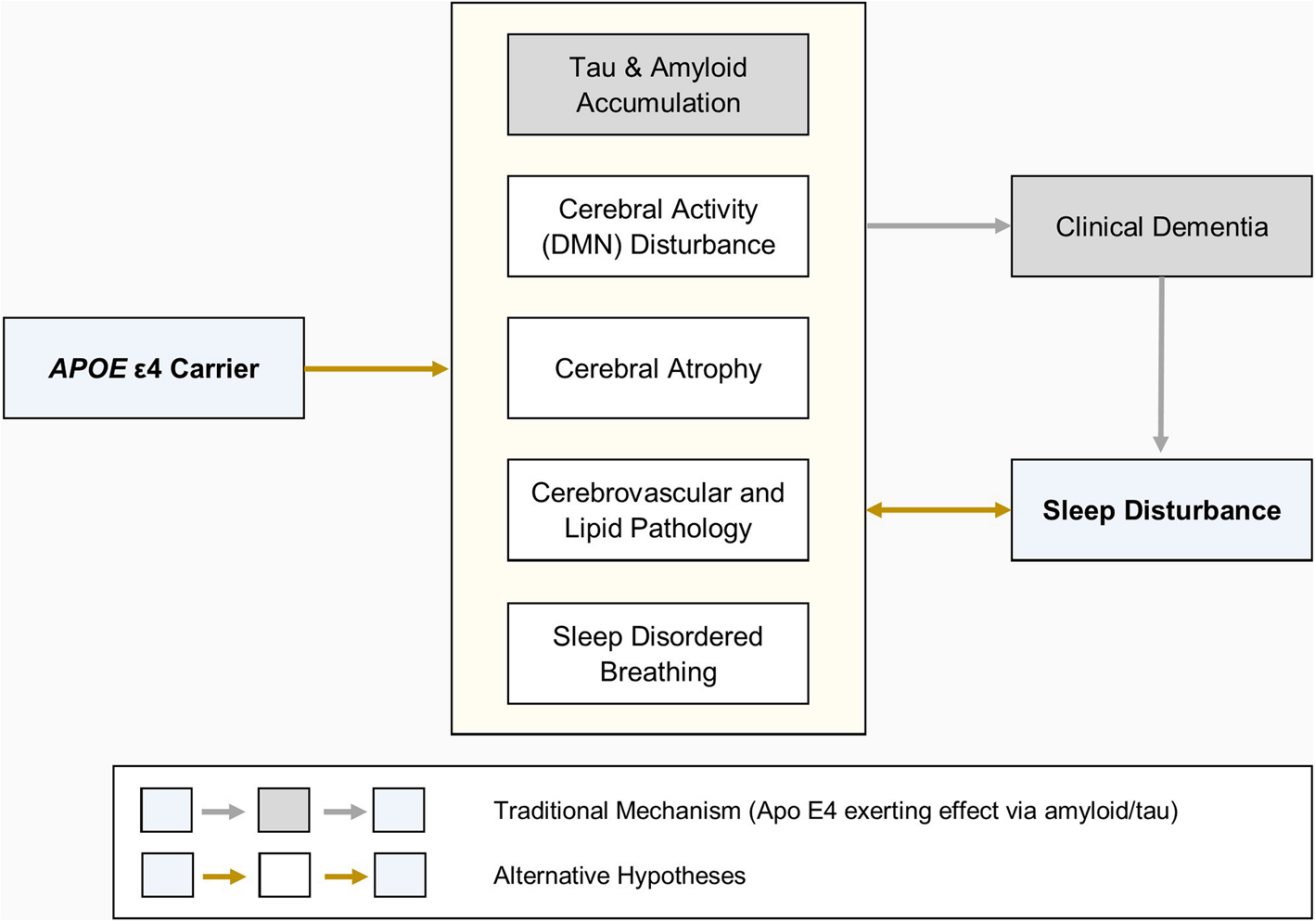
Hypothetical mechanisms of *APOE* ε4-mediated sleep disturbance

*APOE* ε4 is thought to influence AD pathology [9] through enhanced Aβ deposition [61], tau phosphorylation and neurotoxicity [62, 63], all of which may lead to sleep abnormalities [64]. For example, in participants with AD, *APOE* ε4 allele status influences CSF measures of tauopathy, itself associated with night-time behaviour disturbance [65]. We have found that ε4 influences sleep independently of Aβ and tau stage. Hence, as well as its impact on these hallmark features of AD, *APOE* ε4 may also influence AD risk through other neurotoxic pathways and/or loss of neuroprotective functions [9] that have the potential to influence sleep quality (Fig. 3).

### Apo E4-mediated effects on melatonin

In a study of 85 patients with AD, *APOE* ε4 homozygosity compared with heterozygosity was associated with significantly reduced post-mortem CSF melatonin (32pg/ml ± 8 vs 71 ± 7, *p*=0.02) [66]. Reduced melatonin has been linked to sundown syndrome in dementia [67, 68], and replacement improves symptoms according to systematic review [69]. However, melatonin is up to five times higher in healthy adults compared with those with AD [66, 67], and it is plausible that *APOE* ε4 may be associated with reduced melatonin via secondary mechanisms linked to AD severity as opposed to direct effects.

### Apo E4-mediated sleep disordered breathing

*APOE* ε4 was reported to be directly associated with OSA and symptomatic sleep disordered breathing [31], possession of this allele being associated with an approximate doubling of risk for apnoea-hypoxic index > 15 [OR 2.0 (1.2–3.5)] in adults and separately in children [32]. Two main mechanisms for a causal relationship were proposed. Firstly, an ε4-associated increase in respiratory/sleep centre tau or amyloid burden may drive centrally mediated sleep disordered breathing [31]. Alternatively, (or additionally) ApoE4 has a central role in lipid metabolism [70] mediating lipoprotein to cell surface receptor binding, increasing plasma low-density lipoprotein (LDL) levels and accelerating atherogenesis [71]. Centrally mediated sleep disordered breathing is recognised in a wide range of cerebral pathologies [72], including cerebrovascular pathology that might be exacerbated by possession of ε4. A further plausible mechanism, given that *APOE* ε4 predisposes to metabolic syndrome [73] and increased insulin resistance [74] would be through secondary increased obesity; however, ε4 carriers on average have a lower body mass index than do ε3 or ε2 carriers [75, 76]. A systematic review found no support for a causal association between *APOE* ε4 allele and OSA [OR 1.13 (0.86–1.47)] [77], but the authors commented that the studies were heterogeneous, may not have accommodated important gene-gene interactions and may have been underpowered.

### Apo E4-mediated cerebral atrophy

Previous work linked possession of ε4 with accelerated age-related cortical thickness loss [78, 79]. This itself was associated with self-reported sleep disturbance in healthy community dwelling adults [80] and reduced objectively measured total sleep time, random eye movement, N2 and N3 stages of sleep in alcohol-use disorder [81].

### Apo E4 effects on functional cerebral activity

Baseline activity within the Default Mode Network (DMN)—the distributed network of brain regions more active during rβ and characterised by high functional connectivity—is greater in *APOE* ε4 carriers than in non-carriers [82–84]. This overactivity was hypothesised to inhibit brain structures stimulating sleep initiation as described within the ‘failure to inhibit wakefulness’ hypothesis of sleep onset [85, 86]. Whilst the mechanism for this is uncertain, the findings extend to young adults aged 20–35, underlining a potential active role for ε4 outside of established AD pathology [83].

### Study strengths and limitations

This study is subject to several limitations. Firstly, the sample, whilst deeply characterised, was limited by small numbers in the APOE-e4 homozygous group and relatively small numbers within each neuropathological group within the heterozygous group limiting power to detect full effects. Linked to this, p values of ‘statistically significant’ findings were close to 0.05. Reassuringly, however, the sleep disturbance signal was positively correlated with allele number. Participants in the BDR cohort are mostly from less socially and economically deprived parts of the UK [57] and are not therefore fully representative of the general population in the UK. Medication history was not comprehensive enough for inclusion in our analysis and may represent a significant confounder with prescribed sleep medications ameliorating or masking symptoms. Systemic illness that could have impacted on sleep disturbance may not have been detected post-mortem.

The use of the neuropsychiatry inventory sleep disturbance score as principal outcome measure is a further potential weakness. Whilst broad and encompassing a heterogeneous range of disorders, it relies on caregiver report and is therefore potentially subject to bias, e.g., false negative reports of subtle changes. However, it is well-validated, widely used and its reliance on a semi-objective caregiver as opposed to subjective personal reports also has advantages. Outcome scores for this study were obtained within 12 months of death. At the more distant end of this scale, pathological changes could have evolved between clinical data collection and autopsy; however, recall bias from retrospective data collection is eliminated.

Strengths include the categorisation of participants and quantification of AD changes based on the gold standard of post-mortem neuropathology with application of strict exclusion criteria, allowing for the effects of AD pathology to be largely determined in isolation. Data collected as part of the BDR project is standardised and collected as part of a detailed and comprehensive protocol. The population also reflected a range of AD pathology, with 38.2% of the study population recording ABC Scores of ‘Not’ or ‘Low’.

## Conclusion

*APOE* ε4 homozygosity was associated with sleep disturbance, independent of AD pathological change and clinical functional status. Neuropathologically validated clinical studies often provide the first impetus in developing improved understanding of underlying mechanisms of neurological disease. There are a range of plausible mechanisms by which this effect of *APOE* ε4 may be exerted; further systematic testing of which would enhance understanding of sleep disturbance pathways and may subsequently provide treatment targets for this distressing symptom, also linked to AD progression.

## Supplementary Information

**Additional file 1.** Full Population Linear Regression – Additional Models.

## Data Availability

The dataset used and analysed in this current study are available from the first author on reasonable request.

## Abbreviations

AD: Alzheimer’s disease
*APOE*-ε4: Apolipoprotein E epsilon 4
BDR: Brains for Dementia Research
CDR: Clinical Dementia Rating
CDR-SOB: Clinical Dementia Rating–sum of boxes
CNS: Central nervous system
CSF: Cerebrospinal fluid
DMN: Default Mode Network
LB: Lewy body
MMSE: Mini-Mental State Examination
NIA-AA: National Institute on Aging and Alzheimer’s Association
NPI-K: Neuropsychiatry Inventory Measure of Sleep Disturbance
NREM: Non-random eye movement
OR: Odds ratio
OSA: Obstructive sleep apnoea
SD: Standard deviation
TDP-43: Transactive response DNA binding protein–43
UK: United Kingdom
