# Supplementary material for: APOE ε4, Alzheimer’s disease neuropathology and sleep disturbance, in individuals with and without dementia
Source: Alzheimers Res Ther. 2022 Mar 30;14:47. doi: 10.1186/s13195-022-00992-y (PMC8969347; doi:10.1186/s13195-022-00992-y)
Supplement: Supplementary file 1 — Additional file 1. Full Population Linear Regression – Additional Models. [file 13195_2022_992_MOESM1_ESM.docx]

*APOE* ε4, Alzheimer's disease neuropathology, and sleep disturbance, in individuals with and without dementia

SUPPLEMENTARY MATERIALS

**AUTHORS**

Jonathan Blackman, Seth Love, Lindsey Sinclair, Richard Cain, Elizabeth Coulthard

**AFFILIATIONS**

Jonathan Blackman – [jonathan.blackman@nbt.nhs.uk](mailto:jonathan.blackman@nbt.nhs.uk) – North Bristol NHS Trust, Bristol, UK

Seth Love – [seth.love@bristol.ac.uk](mailto:seth.love@bristol.ac.uk) – University of Bristol, Bristol, UK

Lindsey Sinclair – [lindsey.sinclair@bristol.ac.uk](mailto:lindsey.sinclair@bristol.ac.uk) – University of Bristol, Bristol, UK

Richard Cain – [richard.cain@bristol.ac.uk](mailto:richard.cain@bristol.ac.uk) – University of Bristol, Bristol, UK

**Corresponding Author**

Elizabeth Coulthard – [Elizabeth.coulthard@bristol.ac.uk](mailto:Elizabeth.coulthard@bristol.ac.uk) – Learning and Research, University of Bristol, Southmead BS10 5NB

**SUPPLEMENTARY MATERIALS – SECTION 1**

| **SUPPLEMENTARY TABLE 1.** Full Population Linear Regression – Additional Models | | | | | | |
| --- | --- | --- | --- | --- | --- | --- |
| Full Population n=202 | | | | | | |
|  | *APOE* ε4 Heterozygosity | | | *APOE* ε4 Homozygosity | | |
|  | β | SE | p | β | SE | p |
|  |  |  |  |  |  |  |
| Crude Model | 0.67 | 0.54 | 0.221 | 3.18 | 1.20 | 0.008 |
| Model 1  (Adjusted by age, gender and ABC score) | 0.37 | 0.58 | 0.531 | 2.70 | 1.23 | 0.030 |
| Model 2  (Adjusted by age, gender, *APOE* ε2 Status and ABC score) | 0.50 | 0.60 | 0.400 | 2.82 | 1.24 | 0.024 |
| Model 3*  (Adjusted by age, gender, *APOE* ε2 Status, NPI-D (Depression), NPI-E (Anxiety), ABC score and CDR-SOB) | 0.41 | 0.57 | 0.471 | 2.53 | 1.18 | 0.034 |
| Model 4  (Adjusted by age, gender, *APOE* ε2 Status, all NPI domains (NPI-A – NPI-L) and ABC score) | 0.51 | 0.48 | 0.299 | 2.41 | 1.01 | 0.018 |
| Model 5  (Adjusted by age, gender, *APOE* ε2 Status, all NPI domains (NPI-A – NPI-L), ABC score and CDR-SOB) | 0.51 | 0.49 | 0.298 | 2.45 | 1.01 | 0.017 |

*Pre-specified and reported model within main manuscript
